# Multicenter PET image harmonization using generative adversarial networks

**DOI:** 10.1007/s00259-024-06708-8

**Published:** 2024-05-02

**Authors:** David Haberl, Clemens P. Spielvogel, Zewen Jiang, Fanny Orlhac, David Iommi, Ignasi Carrió, Irène Buvat, Alexander R. Haug, Laszlo Papp

**Affiliations:** 1 Division of Nuclear Medicine, Medical University of Vienna, Währinger Gürtel 18-20/E4L, A-1090 Vienna, Austria; 2 Christian Doppler Laboratory for Applied Metabolomics, Medical University of Vienna, Vienna, Austria; 3 LITO Laboratory, U1288 Inserm, Institut Curie, University Paris-Saclay, Orsay, France; 4 Department of Nuclear Medicine, Hospital Sant Pau and Autonomous University of Barcelona, Barcelona, Spain; 5 Present Address: Center for Medical Physics and Biomedical Engineering, Medical University of Vienna, Vienna, Austria

**Keywords:** Harmonization, Quantitative PET, Multicenter, Generative adversarial networks, Deep learning

## Abstract

**Purpose:**

To improve reproducibility and predictive performance of PET radiomic features in multicentric studies by cycle-consistent generative adversarial network (GAN) harmonization approaches.

**Methods:**

GAN-harmonization was developed to harmonize whole-body PET scans to perform image style and texture translation between different centers and scanners. GAN-harmonization was evaluated by application to two retrospectively collected open datasets and different tasks. First, GAN-harmonization was performed on a dual-center lung cancer cohort (127 female, 138 male) where the reproducibility of radiomic features in healthy liver tissue was evaluated. Second, GAN-harmonization was applied to a head and neck cancer cohort (43 female, 154 male) acquired from three centers. Here, the clinical impact of GAN-harmonization was analyzed by predicting the development of distant metastases using a logistic regression model incorporating first-order statistics and texture features from baseline ^18^F-FDG PET before and after harmonization.

**Results:**

Image quality remained high (structural similarity: left kidney $\ge$ 0.800, right kidney $\ge$ 0.806, liver $\ge$ 0.780, lung $\ge$ 0.838, spleen $\ge$ 0.793, whole-body $\ge$ 0.832) after image harmonization across all utilized datasets. Using GAN-harmonization, inter-site reproducibility of radiomic features in healthy liver tissue increased at least by $\ge$ 5 ± 14% (first-order), $\ge$ 16 ± 7% (GLCM), $\ge$ 19 ± 5% (GLRLM), $\ge$ 16 ± 8% (GLSZM), $\ge$ 17 ± 6% (GLDM), and $\ge$ 23 ± 14% (NGTDM). In the head and neck cancer cohort, the outcome prediction improved from AUC 0.68 (95% CI 0.66–0.71) to AUC 0.73 (0.71–0.75) by application of GAN-harmonization.

**Conclusions:**

GANs are capable of performing image harmonization and increase reproducibility and predictive performance of radiomic features derived from different centers and scanners.

**Supplementary Information:**

The online version contains supplementary material available at 10.1007/s00259-024-06708-8.

## Introduction

Positron Emission Tomography (PET) has established itself as a valuable tool in cancer diagnosis, prognosis, and clinical treatment decision-making [1–3]. With the upcoming shift from qualitative to quantitative imaging, hopes have been raised that the precise quantification of image-derived biomarkers will extend the current capabilities of PET, and thus, improve patient outcomes. Several studies on quantitative PET show promising results for tumor texture analysis, indicating the possibility of capturing tumor heterogeneity using radiomic features and the subsequent utilization of these features for the prediction of clinical outcomes [4, 5]. Radiomics refers to an approach that extracts and analyzes a large set of quantitative image-derived features (e.g., intensity, shape, texture) that are used to reveal associations between medical imaging data and patient outcomes. Moreover, deep learning approaches are rapidly gaining relevance in PET imaging, enabling automated lesion segmentation [6, 7], classification and detection of disease patterns [8, 9]. However, the clinical translation of these methods is currently lacking, which can be partly attributed to the low robustness and poor generalizability of these approaches. It has been shown that most radiomic feature values are sensitive to different scanners [10], acquisition protocols [11], and reconstruction settings [12]. This further applies to deep learning-based methods, where such acquisition shifts cause poor generalization to new unseen data [13, 14]. Yet, this reflects current clinical practice since ever-evolving imaging aspects such as scanners and protocols cannot be entirely standardized. The diversity of image acquisition leads to scans with different styles (e.g., caused by different scanners) and textures (e.g., induced by different reconstruction algorithms) among sites. We define *style* as the global visual appearance of an image influenced by factors such as image contrast or brightness, and *texture* as a local characteristic related to the spatial distribution of voxel intensities.

In response, we propose a deep learning-based PET image harmonization method (GAN-harmonization) that aims to harmonize PET scans acquired from different centers and scanners. Our objective is to improve the reproducibility and predictive performance of quantitative image biomarkers. We utilize a cycle-consistent generative adversarial network (CycleGAN) that performs image style and texture translation between unpaired PET scans from different centers and scanners. The approach is—in contrast to existing feature-based PET harmonization methods [15, 16]—purely image-based, which allows physicians and researchers to have access to the images after harmonization. This enables the potential use of the harmonized images in subsequent downstream tasks such as deep learning image segmentation and classification. We evaluate GAN-harmonization by applying it on two different datasets and tasks. First, we perform image harmonization on a dual-center whole-body lung cancer (LC) dataset where we investigate the reproducibility of radiomic features in healthy liver tissue before and after harmonization. Second, we apply GAN-harmonization to a head and neck (HN) cancer dataset acquired from three centers, where we analyze the clinical impact by predicting patient outcome. No harmonization strategy at all and the widely used feature-based harmonization technique ComBat served as benchmarks.

## Materials and methods

### Datasets

#### Lung cancer

Patients from two different centers with histologically proven non-small cell lung cancer (NSCLC) were retrospectively collected. The dataset consisted of 168 patients (65 female, 103 male, 66 ± 9 years) from the University Hospital Tübingen as part of the open access autoPET challenge dataset [17] and 97 patients (62 female, 35 male, 63 ± 8 years) from University Hospital of Budapest as part of a retrospectively collected patient cohort (2009–2021). For both cohorts, inclusion criteria were histologically confirmed diagnosis of NSCLC. Furthermore, in the Budapest cohort, eligibility for surgical tumor resection was an additional requirement. Institutional review board approval was obtained for the Budapest cohort, and the requirement to obtain informed consent was waived (ID 1649/2016). All patients underwent whole-body ^18^F-FDG PET/CT imaging. As no clinical outcome data was available for this dataset, it was primarily used to demonstrate the capability of the GAN to process whole-body scans and to analyze the effect of GAN-harmonization on healthy liver tissue, as it is the recommended reference region by the European Association of Nuclear Medicine (EANM) for quality control purposes [18].

#### Head and neck

PET/CT studies and clinical outcome data from 197 patients from three centers in Québec (Canada) with histologically proven head and neck squamous cell carcinoma (HNSCC) were retrospectively collected from The Cancer Imaging Archive [19, 20]. The endpoint of interest was defined as the development of distant metastases (DM). This endpoint was chosen because Vallières et al. showed in their original study that radiomic features derived solely from PET had the highest predictive value for this particular endpoint among all others investigated [21]. The dataset included 65 patients (16 female, 49 male, 63 ± 9 years, 3 DM) from the Centre hospitalier de l’Université de Montréal (CHUM), 91 patients (17 female, 74 male, 61 ± 11 years, 16 DM) from the Hôpital général juif (HGJ), and 41 patients (10 female, 31 male, 67 ± 9 years, 11 DM) from the Hôpital Maisonneuve-Rosemont (HMR). The comprehensive clinical characteristics of these cohorts have been published [21]. All patients underwent pre-treatment ^18^F-FDG PET/CT imaging with a field of view covering only the head and neck region. This dataset was used to assess the clinical impact of GAN-harmonization by investigating the ability of a radiomic model to perform well on data from different centers. Note that due to the limited field of view of this dataset (no abdominal organs visible in PET/CT such as the liver), the effect of GAN-harmonization on healthy liver tissue (as conducted in the lung cancer dataset) could not be performed. Site-specific PET acquisition characteristics for both datasets are listed in Table 1.

**Table 1:** PET/CT acquisition characteristics of each dataset and center

Dataset	Center	PET/CT scanner	Reconstruction	Scans	Matrix size	Voxel size (mm^3^)
Lung	TUB	Siemens Biograph mCT	OSEM (2i21s) 2 mm Gaussian kernel	168	400 × 400	2.04 × 2.04 × 3.00
	BUD	GE Discovery IQ	VUE Point HD† SharpIR	53 44	168 × 168 256 × 256	4.07 × 4.07 × 3.00 2.73 × 2.73 × 3.26
Head and neck	CHUM	GE Discovery STE	OSEM†	43 17 5	144 × 144 128 × 128 128 × 128	4.00 × 4.00 × 4.00 3.52 × 3.52 × 3.27 5.47 × 5.47 × 3.27
	HGJ	GE Discovery ST	OSEM†	90 1	128 × 128 128 × 128	3.52 × 3.52 × 3.27 4.69 × 4.69 × 3.27
	HMR	GE Discovery STE	OSEM†	30 11	128 × 128 128 × 128	3.52 × 3.52 × 3.27 5.47 × 5.47 × 3.27

*TUB* University Hospital Tübingen, *BUD* University Hospital of Budapest, *CHUM* Centre hospitalier de l’Université de Montréal, *HGJ* Hôpital général juif, *HMR* Hôpital Maisonneuve-Rosemont

^†^Number of iterations (i) and subsets (s) were not reported

### Deep learning-based PET image harmonization

**Fig. 1:**
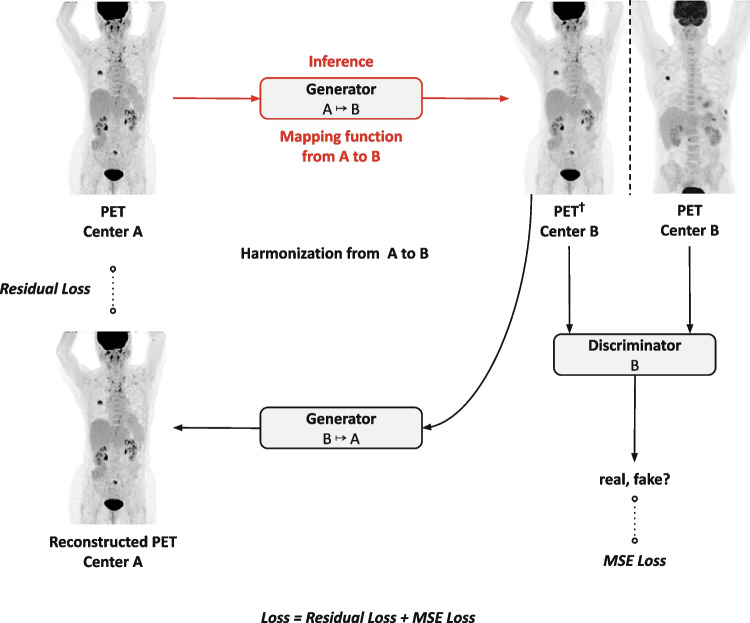
Schematic overview of the training and inference process for the harmonization from an arbitrary center A to center B. An arbitrary PET scan from center A is fed to the generator network G: A ↦ B that is trained to learn a mapping function between center A and B. The goal here is to generate an image that is conditioned to the reference center B, and hence, sampled from their underlying distribution. The generator outputs PET^†^ in domain B, i.e., the input image with style and texture from the reference center B. A discriminator network is directly coupled to the output and trained to distinguish between real PET images from center B and synthetically created PET images from the generator G: A ↦ B**.** By doing so, the generator G: A ↦ B is forced to generate high quality synthetic imaging data by fooling the discriminator. Global clinically relevant anatomical structures are retained by the utilization of a second generator network G: B ↦ A, with the ultimate goal to ensure identity when performing the inverse transformation (cycle consistency). At test time (red), the trained generator network G: A ↦ B is used to transform images from center A to center B. Dotted lines indicate loss function measurements. The entire loss is the sum of the adversarial loss and the cycle-consistency loss

A cycle-consistent generative adversarial network (CycleGAN) was developed to harmonize PET images among multiple centers and scanners. A CycleGAN architecture was chosen over other GAN architectures as it is particularly suited for tasks where paired data is not readily available. Paired data would require the same patient to be imaged at both sites in a short time interval (so that no changes in the disease stage occur) and in similar conditions (e.g., similar blood glucose levels). CycleGANs perform unpaired image-to-image translation, allowing it to learn the mapping between different centers without requiring explicit correspondence between the images. The originally published CycleGAN architecture [22] was modified by adding the capability of processing volumetric images to better deal with the 3D nature of tomographic biomedical images. This was achieved by patch-wise training and a sliding window inference. Moreover, nine residual blocks in the generator networks were used, and the cycle-consistent loss function was adapted to represent the residual error between the input and the generated output image. A schematic overview of the training and inference process is shown in Fig. 1. Required image preprocessing steps, the network architecture of each component, and training details are described in Supplementary Material A, following the CLAIM guidelines [23].

### Evaluation

Two different GAN models were trained, one for each dataset. Training sets were ensured to be properly stratified with respect to the center and scanner (imaging protocol). For the lung cancer dataset, the training set was split with regard to the center (A: Budapest, B: Tübingen). For the head and neck dataset, the three centers were partitioned based on their scanners and imaging protocols (A: HGJ, B: CHUM-HMR). Center CHUM and HMR were merged and processed as a combined center CHUM-HMR. This step was performed as center CHUM and HMR utilized the same scanner and imaging protocol (Table 1). We used the exact same stratifications to compare GAN-harmonization with the feature-based harmonization method ComBat [15, 24]. All experiments were performed with each site serving once as the reference site (harmonization from A to B and vice versa).

#### Image quality

To evaluate the accuracy of the GAN to generate realistically looking harmonized images, we calculated voxel-wise image quality metrics between the original input images and the GAN-harmonized output images including the structural similarity index measure (SSIM), normalized root mean squared error (NRMSE), and the peak signal-to-noise ratio (PSNR). These metrics were used as an indicator for cycle consistency and are described in detail in Supplementary Material B. For both datasets, metrics were calculated on a global level using a body mask. They were semi-automatically generated in a two-step process, first a threshold of 0.1 SUV based on physical considerations and previous experiments was used followed by morphological operations to create an initial rough body mask, and subsequently validated and corrected by a physician. This step was performed to suppress unnecessary background voxels, which could conflate the metrics to be overly optimistic. In addition, for the LC dataset, metrics were also calculated locally for different target organs (left kidney, right kidney, liver, lung, spleen). Organ segmentations were derived using TotalSegmentator [25] and validated by a physician. Patients with missing organs were excluded (*n* = 1: Hungary, *n* = 4: Tübingen).

#### Reproducibility analysis of radiomic features

All patients with healthy liver tissue were selected by a physician. Healthy liver tissue was defined as having no evidence of metastases or other pathologies in the liver as determined from the PET/CT imaging data by nuclear medicine physician with 7 years of experience. Fifty subjects with healthy liver tissue were then randomly selected at each site for the evaluation of the GAN-harmonization on the LC dataset. For each subject, a spherical volume of interest (VOI) with 3-cm diameter was placed in the upper right lobe of the liver, following the latest EANM procedure guideline version 2.0 [18]. The coefficient of variation (COV = $\sigma /\mu$) was calculated from this VOI and compared between the two centers.

Moreover, 93 IBSI-1 compliant radiomic features (first-order statistics and texture, see Supplementary Table 1) were computed from the same VOI using PyRadiomics [26]. The effect of GAN-harmonization on radiomic feature distributions was evaluated by measuring the feature overlap coefficient. The feature overlap is a similarity measure and quantifies how much the feature distributions from two different sites overlap with each other (graphical representation in Supplementary Fig. 2). The overlap is quantified by calculating the overlapping area between the two distributions [27, 28]. This approach was chosen as it allowed to compare distributions of unequal sizes (different number of patients at each site) and because similar approaches have been used in other harmonization studies [15, 29]. An overlap of 1 implied perfect agreement between the two distributions and consequently high reproducibility, while an overlap of 0 corresponded to perfect disagreement and low reproducibility across the two sites.

#### Clinical outcome prediction

The effect of image harmonization on a given clinical endpoint was evaluated by building a logistic regression model to predict the development of DM (binary classification: yes, no) based on radiomic features (first-order statistics and texture, see Supplementary Table 1) extracted from the largest tumor lesion in the baseline PET scan. Tumor delineations were reused from the originally published study [21] and available as part of the open-access repository. The same delineations were used for the harmonized and unharmonized images. We also assessed the performance of the same classifier in an identical setting after *(i)* feature harmonization with ComBat [15] and *(ii)* after both image and feature harmonization. ComBat was performed for each feature separately and without any parametric adjustments or covariate.

For the clinical outcome prediction, patients were randomly assigned (without replacement) to a training (80%, 157 patients) and test (20%, 40 patients) dataset. The split was performed in a stratified manner to ensure the preservation of relative class frequencies. This procedure was repeated 100-times, effectively ending up with a 100-fold Monte Carlo cross-validation as recommended [30]. All experiments were performed with identical fold configurations, thus, ensuring comparability between the different approaches. No feature selection or dimensionality reduction technique was used. The rationale behind this decision was to set up a scenario where the effect of harmonization on a model can be accurately measured. Utilizing feature selection might lead to the selection of features that might not benefit from harmonization. Consequently, such an approach would hinder an accurate measurement of the impact of harmonization on the model. This rationale was in line with the aim of the evaluation, i.e., not to build a new state-of-the-art HNSCC prediction model, but rather to evaluate the effect of harmonization on a given clinical endpoint. As a mitigation strategy for the added risk of overfitting, we utilized robust cross-validation. The area under the receiver operating characteristic curve (AUC) was used as the primary classifier performance metric.

#### Statistical analyses

The Shapiro–Wilk test was used to test for normality of the data. If normality was not given, the Wilcoxon signed-rank test was used for comparison of all paired variables, while the Mann–Whitney *U* test was used for independent variables. For normally distributed data, Student’s *t* test was performed. For the comparison of AUCs, the DeLong test was used. Results were considered statistically significant if the two-sided *P* value was less than 0.05. Statistical analysis was performed with the SciPy Python package 1.10.0.

## Results

### GAN performance

**Fig. 2:**
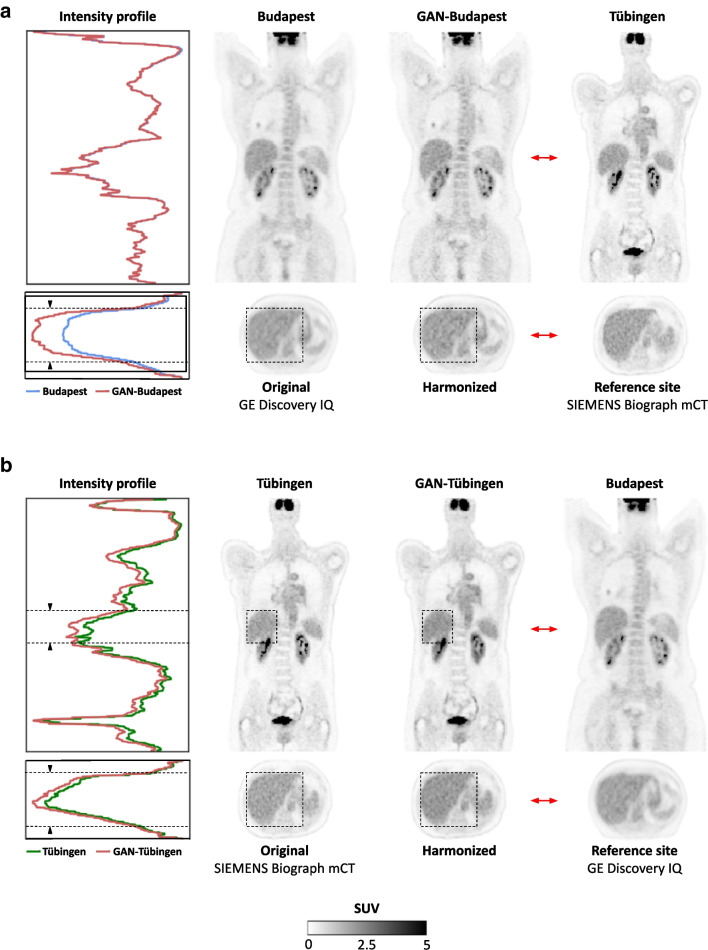
Qualitative results from the LC dataset. The first column shows the intensity profile of the original PET scan before harmonization (second column) and the output of the GAN after performing harmonization (third column). While the overall global structure was not altered by the GAN (second and third column), style and texture were adapted to the reference site (fourth column – we show here a randomly selected patient from the reference site as a representative). Differences in texture between the original and the GAN output are highlighted with dashed lines (first-third column). The red arrow indicates pairs of images with similar style and texture after harmonization. PET scans are shown for the harmonization from **a** Budapest to Tübingen and **b** Tübingen to Budapest. Note that there were no paired patients among the two centers

**Fig. 3:**
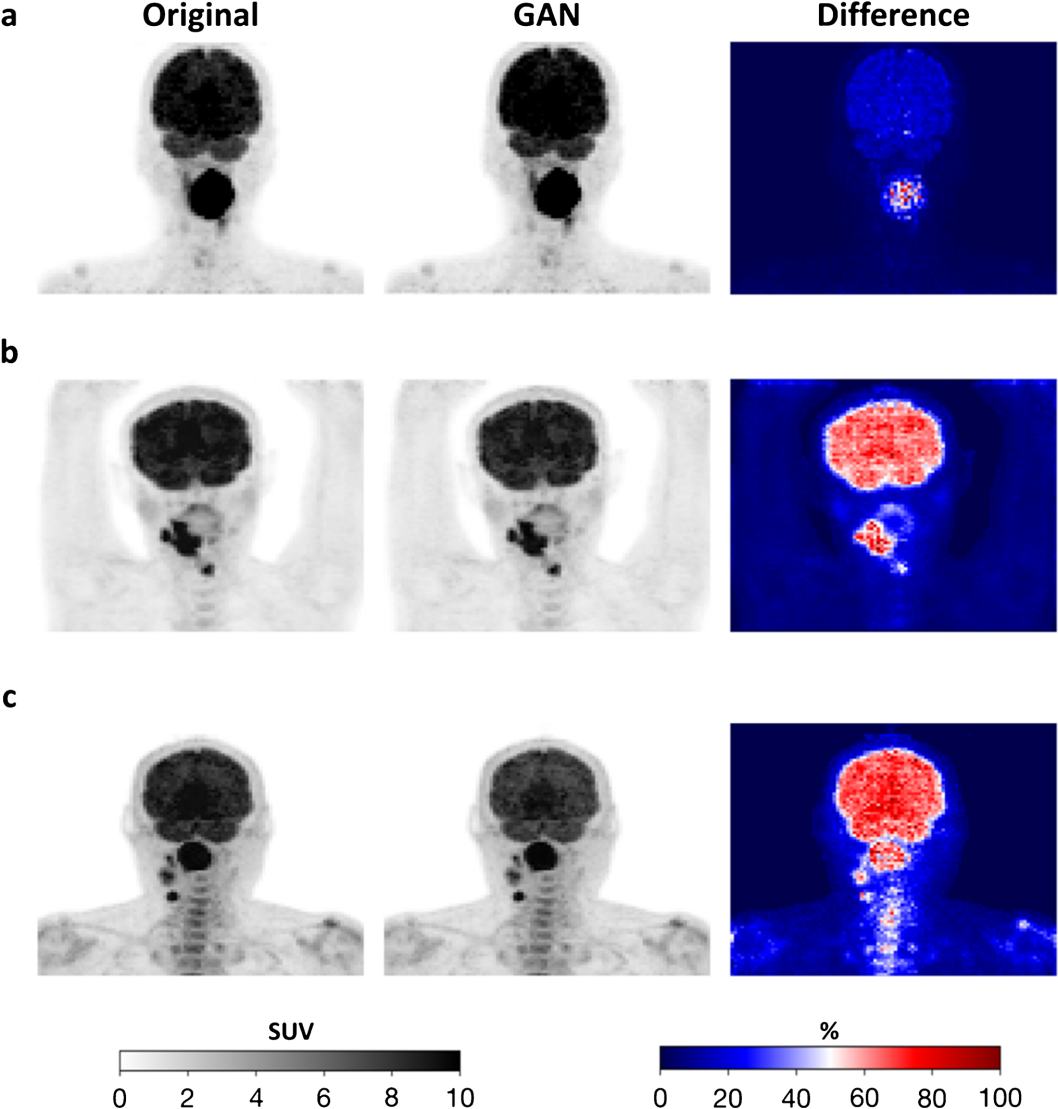
Representative examples (maximum intensity projections) before and after GAN-harmonization. The columns (from left to right) represent the original input images, GAN-harmonized output images, and their voxel-wise percent difference maps, indicating which part of the image was changed by the GAN. **a** HGJ to CHUM-HMR, **b** + **c** CHUM-HMR to HGJ. The patient in panel **a**) shows only changes in the tumor region compared to the patients in panel **b**) and **c**) which may be explained by a higher uptake in the lesion compared to the brain

Qualitative results for a representative example from each site are shown in Fig. 2 (LC dataset) and Fig. 3 (HN dataset). The direct head-to-head comparison between the unharmonized (Fig. 2, second with fourth column) and harmonized (Fig. 2, third with fourth column) PET shows visual differences in the liver uptake before the harmonization, which were minimized and adapted to match the reference site after performing GAN-harmonization.

For the HN cancer cohort, three representative examples from each site are shown before and after harmonization along with their voxel-wise percent difference map (Fig. 3, third column). The residual images highlight regions that were mostly affected by the GAN-harmonization and suggest that style and texture translation occurred predominantly in high uptake regions such as in brain or lesions.

**Fig. 4:**
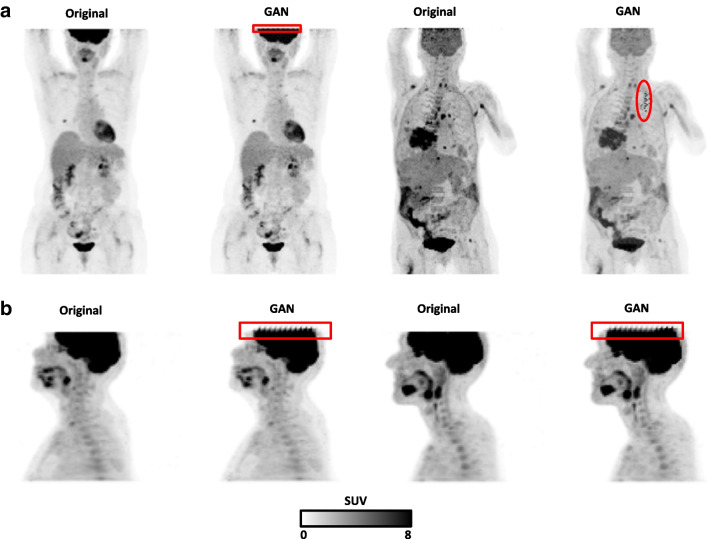
Selected examples (maximum intensity projections) before and after GAN-harmonization containing artifacts induced by the GAN. Images are sampled from **a** the lung cancer dataset and **b** the head and neck cancer dataset

Representative examples with GAN induced image artifacts are shown in Fig. 4 for both datasets. Minor artifacts were observed in the noisy marginal slices of the PET scan (first few slices in the brain) in 11/197 (5.6%) scans. In 1/265 (0.4%) scans an image artifact in the upper right thorax region (Fig. 4a, fourth column) was present.

**Fig. 5:**
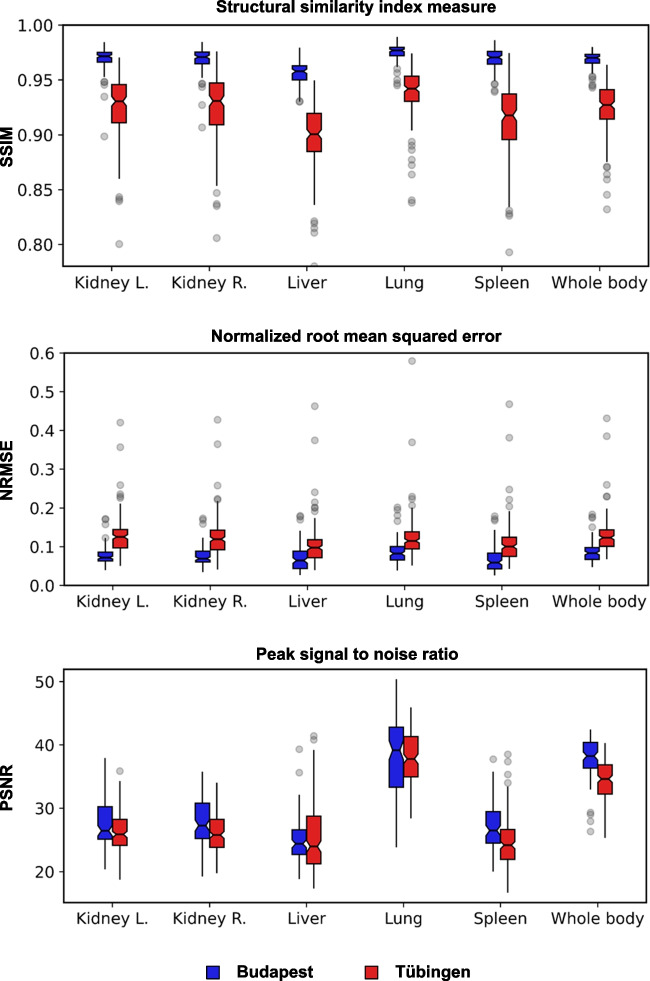
Quantitative results for image similarity and image quality metrics between the original input images and their GAN-harmonized counterparts. Data are reported as the median (center line) ± interquartile range (box edges). Whiskers are 1.5 times the interquartile range. Data points outside the whiskers are considered outliers

Quantitative results for image quality metrics are presented in Fig. 5 for the entire body and different organs of the whole-body LC dataset. The results for the HN dataset are shown in Supplementary Table 2. The overall high SSIM and low NRMSE indicated that no global anatomical and metabolic structures were altered in the images.

### Impact on reproducibility of radiomic features

**Fig. 6:**
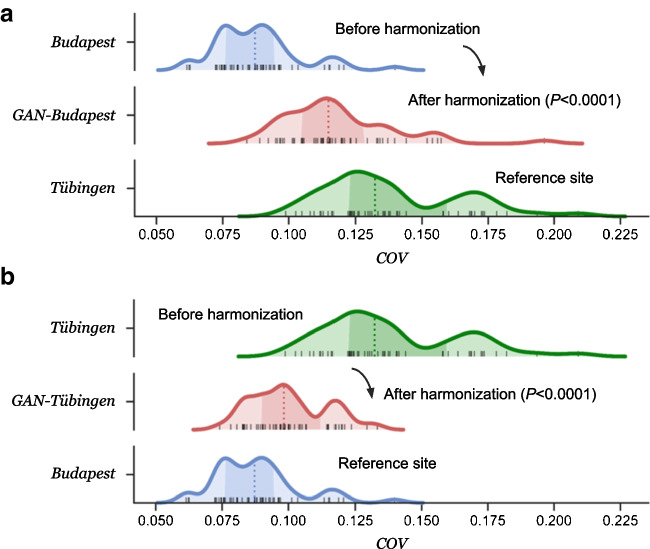
Coefficient of variation (COV) measured in healthy liver tissue of the right upper lobe from fifty randomly selected subjects at each site. The distribution of the COV is shown before and after harmonization for each site. Prior to harmonization, the COVs between the two sites (Budapest, Tübingen) are not well aligned, indicating site-specific differences in the images. Those differences were substantially reduced after harmonization (GAN-Budapest, GAN-Tübingen), resulting in a better alignment and higher spatial congruity when compared to their reference site. The *P*-values are presented for the comparison of the distances between the two distributions to the reference site. Harmonization from **a** Budapest to Tübingen and **b** Tübingen to Budapest

The median COV was 0.09 (interquartile range IQR: 0.08–0.09) for Budapest and 0.13 (IQR: 0.12–0.16) for the Tübingen cohort. The distribution of COVs was significantly different (*P* < 0.0001) between the two centers. After performing GAN-harmonization, the median COV was 0.11 (IQR: 0.10–0.13) for GAN-Budapest and 0.10 (IQR: 0.09–0.11) for GAN-Tübingen. The distance between the COV distributions was significantly (*P* < 0.0001) reduced after GAN-harmonization, regardless of the reference site selection. A visual presentation of the results is shown in Fig. 6.

**Fig. 7:**
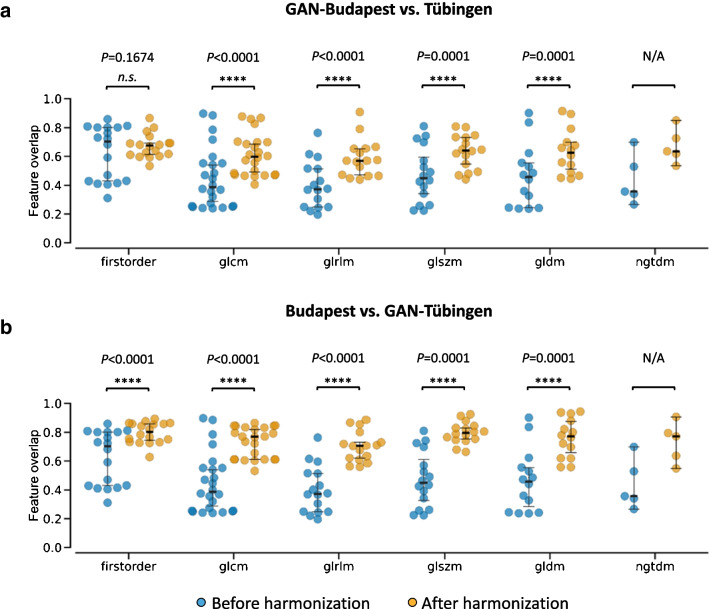
Radiomic feature overlap before (blue) and after (orange) GAN-harmonization. Groupwise comparison of the feature overlap before (left, blue) and after (right, orange) harmonization for each feature class separately. *P* values are based on Wilcoxon signed-rank test. No statistical test was performed for NGTDM features due to their low sample size (*n* = 5). Harmonization from **a** Budapest to Tübingen and **b** Tübingen to Budapest. First-order first-order statistics, GLCM Gray Level Cooccurrence Matrix, GLRLM Gray Level Run Length Matrix, GLSZM Gray Level Size Zone Matrix, GLDM Gray Level Dependence Matrix, NGTDM Neighboring Gray Tone Difference Matrix

A significant improvement in reproducibility was observed for all feature classes after harmonizing Tübingen to Budapest (Fig. 7b). For the inverse harmonization direction (Budapest to Tübingen; Fig. 7a), first-order statistics features were not significantly affected by the harmonization (*P* = 0.1674; 5 ± 14% improvement on average). Local radiomic feature reproducibility was generally higher when selecting Budapest as the reference site. The absolute improvement per feature class is summarized in Table 2.

**Table 2:** Absolute improvement in inter-center reproducibility per feature class. Data are percentages of the average increase (± one standard deviation) of the radiomic feature distribution overlap between the two centers

Feature class	Feature count	GAN-Budapest vs. Tübingen	Budapest vs. GAN-Tübingen
First-order	18	5 ± 14	17 ± 12
GLCM	24	16 ± 7	28 ± 13
GLRLM	16	19 ± 5	30 ± 8
GLSZM	16	16 ± 8	32 ± 15
GLDM	14	17 ± 6	30 ± 10
NGTDM	5	23 ± 14	29 ± 21

*First-order* first-order statistics, *GLCM* Gray Level Cooccurrence Matrix, *GLRLM* Gray Level Run Length Matrix, *GLSZM* Gray Level Size Zone Matrix, *GLDM* Gray Level Dependence Matrix, *NGTDM* Neighboring Gray Tone Difference Matrix

### Impact on clinical outcome prediction

In total, we compared three different harmonization strategies: (1) the proposed image-based GAN-harmonization, (2) the feature-based ComBat technique, and (3) a joint approach of GAN image harmonization followed by ComBat feature harmonization. For comparison, we also calculated the performance of a baseline model, where no harmonization strategy was employed.

In Table 3, the approaches are compared in terms of their average test AUC over a 100-fold Monte Carlo cross-validation. The baseline model achieved a cross-validated test AUC of 0.68 (95% confidence interval CI: 0.66–0.71) for the prediction of DM. The proposed GAN-harmonization approach improved classification performance significantly by + 4% (*P* = 0.0001) for the harmonization of CHUM-HMR to HGJ and by + 5% AUC (*P* < 0.0001) for HGJ to CHUM-HMR, resulting in AUC 0.72 (95% CI: 0.70–0.74) and AUC 0.73 (95% CI: 0.71–0.75), respectively. The results suggested no dependency regarding the selection of the reference site. No significant impact on the classification performance was found for feature harmonization with ComBat (CHUM-HMR to HGJ: *P* = 0.1787; HGJ to CHUM-HMR: *P* = 0.1463) when compared with the baseline model. The most contributing features for each harmonization approach are listed in Supplementary Table 3 (reference site: HGJ) and Supplementary Table 4 (reference site: CHUM-HMR).

**Table 3:** Areas under the receiver operating characteristic curves (AUCs) for the clinical outcome prediction (development of distant metastases at a later time point). Data are presented as mean values and 95% confidence interval (CI) over 100-fold Monte Carlo cross-validation and are reported for the test set. The reference site denotes the site to which the harmonization was performed. Statistical significance was assessed by comparing the AUCs of each method with the baseline (unharmonized) results using the DeLong test

	Harmonization method	AUC	95% CI	*P* value
Baseline	None	0.68	0.66–0.71	-
Reference site: HGJ	GAN	0.72	0.70–0.74	0.0001
	ComBat	0.69	0.67–0.72	0.1787
	GAN and ComBat	0.71	0.69–0.73	0.0046
Reference site: CHUM-HMR	GAN	0.73	0.71–0.75	< 0.0001
	ComBat	0.69	0.67–0.72	0.1463
	GAN and ComBat	0.74	0.72–0.76	< 0.0001

The joint approach revealed classification performances of AUC 0.71 (95% CI: 0.69–0.73, + 3% compared to baseline, *P* = 0.0046) for the harmonization of CHUM-HMR to HGJ and AUC 0.74 (95% CI: 0.72–0.76, + 6% compared to baseline, *P* < 0.0001) for HGJ to CHUM-HMR. The predictive performance of the joint model was significantly better when comparing the baseline with the joint model. However, no evidence for differences in predictive performance were observed between the GAN-only and the joint approach (CHUM-HMR to HGJ: *P* = 0.1184; HGJ to CHUM-HMR: *P* = 0.2662) within this experiment.

The voxel-wise percent difference maps in Fig. 3 column 3 show that distinct changes in the PET occurred in lesions, which in turn were the inputs for the prediction model, as the radiomic features were computed from these regions. The improvement in the classification performance was directly associated with changes that were caused by the GAN-harmonization.

## Discussion

Quantitative PET imaging has become a promising method that provides additional information regarding prognosis and treatment response monitoring in cancer patients which goes beyond traditional qualitative imaging. However, the sensitivity of quantitative imaging markers to different scanners, acquisition protocols, and reconstruction algorithms is a limiting factor in large-scale multi-institutional studies. To bridge this gap, we developed a deep learning-based image harmonization method relying on CycleGANs that normalize PET scans to remove site-specific image characteristics while retaining the clinically relevant biological information for the prediction of distant metastases in HN cancer patients. This was evidenced by high image similarity measures after harmonization and the preservation of predictive performance in a classification downstream task. We demonstrated the ability of CycleGANs to generate and model high quality (whole-body) PET scans that are drawn from a given reference data distribution. Moreover, we have observed an increase in reproducibility of radiomic features after applying GAN image harmonization and showed that harmonized data enables building higher performing models (based on cross-validation) from multi-center data compared to models that were built from non-harmonized data.

Substantial harmonization efforts have been made towards prospective studies eventually resulting in the EARL guidelines [18, 31]. However, these guidelines rely on phantom data and hence may not be applicable in retrospective studies which are needed to accelerate clinical translation. Post reconstruction feature-based harmonization methods relying on ComBat [32] have been proposed and successfully used in radiomics studies [15, 29, 33]. However, ComBat cannot be used in deep learning applications that operate on an image level (e.g., image segmentation), demanding the need for an image-based solution. In contrast to simple image smoothing techniques that typically rely on predefined filters or heuristics, CycleGANs utilize deep neural networks and are hence capable of learning a mapping function between a source and a target center. They can capture complex relationships that may exist between the imaging data. Moreover, they aim to preserve the diagnostic information present in the original images through the cycle-consistency loss. This constraint is missing for simple image filters, and the application of such may smooth out important features, and thus, potentially interfere with the underlying biological signal.

The capabilities of deep generative models to perform image harmonization have been studied for different imaging modalities [34–39]. For brain magnetic resonance imaging (MRI), Hognon et al. proposed a contrastive deep image adaptor network, showing a positive impact of their method on a downstream segmentation task [34]. In their approach, the authors used a combination of several different loss functions to train the network. Tixier et al. conducted a comparative study between conventional histogram matching and generative adversarial networks when being used for radiomics data harmonization in outcome prediction modeling [35]. They found that the predictive value of certain radiomic features could be recovered after applying multi-institutional harmonization and showed that GAN-harmonization outperformed histogram matching. The influence of image harmonization on generalizability of a radiomics model in grading meningiomas on external validation has been studied by Park et al. [36]. The variability of radiomic features in chest radiography acquired from two different vendors was evaluated previously [37]. Both studies showed that a CycleGAN can reduce image variability while improving the predictive performance of radiomics features, which is in line with our study. Similar to this study, Marcadent et al. [37] reported high structural similarity measures between the unharmonized and harmonized images and an increase in feature reproducibility after performing GAN-based texture-translation. Choe et al. presented a deep learning-based image conversion approach which effectively reduced radiomic feature differences caused by different reconstruction kernels in chest CT imaging for pulmonary nodules or masses [38]. However, besides the varying imaging modalities and clinical tasks, all studies used a 2D input, which is different from our work. While the loss of spatial information along the z-axis is not a constraint for chest radiography, which is inherently a planar imaging technique, it can be difficult for tomographic imaging such as PET. The utilization of 3D convolutions for volumetric images and objects is not only more intuitive, but it also adds additional contextual information to the network. Especially in whole-body imaging, where many structures cannot be recognized from a single slice, information transfer between adjacent slices is beneficial to not cause inter-slice artifacts that may adversely affect the performance as shown in previous studies [40]. By successfully applying GAN-harmonization to a whole-body lung cancer PET dataset and being able to produce high quality images, we have shown evidence for the extended use of GANs for whole-body imaging, enabling the potential application to other modalities (CT or MRI), as demanded by others [41].

It is important to note that our study had limitations that should be taken into consideration. Although we observed overall high global image similarity, we identified potential failure modes of the GAN in regions with varying field of views within the datasets as typically present in the head and brain regions. GAN predictions for those regions exhibited higher uncertainties but may be regularized by enlarging the training dataset with a diverse set of samples. GAN-induced image artifacts require visual inspection by a physician and potentially confound the uptake values in the corresponding regions. Moreover, larger datasets from different centers and scanners are needed to further investigate the ability of GAN-harmonization to improve generalizability of radiomics and deep learning models for different applications and diseases. This is particularly important in a more clinically realistic scenario and with large external holdout cohorts. Moreover, due to the relatively small and imbalanced HN dataset, predictive performance measurements were performed by mixing all three centers in a 100-fold Monte Carlo cross-validation rather than training the radiomics model on one center and deploying it on the others and vice versa. We have chosen this evaluation strategy in favor of having a higher statistical power due to the cross-validation scheme. Furthermore, no feature selection was used since we wanted to truly assess the contribution of all individual features after harmonization. In the HN dataset, tumor delineations were taken over from [21] and therefore based on the unharmonized images. The same delineations were used for the harmonized images. Even though this procedure ensures no bias towards inter and intra-operator variability, it does not reflect an ideal clinical scenario, in which tumor delineations should have been drawn on the harmonized images. Radiomic features were extracted from the largest lesion only. This procedure was performed as there is currently no clear consensus of how to aggregate features from multiple lesions [42] and previous studies focused on the largest lesion [43, 44]. Even though this study did not aim to build the best possible prediction model for HNSCC, it is suboptimal and potentially weakening the results. All these factors may make the results of the HN outcome prediction overoptimistic, and it cannot be guaranteed that GAN-harmonization made overfitting of the model easier. It is also important to note that the experimental setup for the HN outcome prediction did not meet the optimal conditions for using ComBat, as ComBat typically requires each batch to include a sufficient number of patients (around 20–30) acquired with a single imaging protocol on the same scanner. Moreover, the varying numbers of patients who developed DM (CHUM: *n* = 3, HGJ: *n* = 16, HMR: *n* = 11) of each center indicate clinical differences within the HN subcohorts, violating the assumption of ComBat that the different samples come from the same population and are affected by technical differences only. Additionally, a covariate accounting for the different voxel and matrix sizes within each center (Table 1) could have been introduced to both ComBat and GAN for improved harmonization results. This was not carried out in our experiments, because it is not recommended to use covariates for ComBat when having less than 20–30 patients per covariate in each batch [45]. This might explain the results obtained with ComBat in the context of this study. Finally, all harmonization methods are inherently and by design static, meaning that they require access to the data from both sites at once. Similar to ComBat, which needs to be performed for each new dataset, GAN-harmonization must therefore be explicitly retrained on a dataset-by-dataset basis if acquisition shifts occur. This process is computationally more expensive compared to the less complex ComBat method. At the time the GAN is trained and in deployment, it cannot cope with situations where acquisition shifts occur at unknown timepoints, e.g., when an additional new scanner is introduced, or protocols change. While this assumption can hold true in well-defined retrospective (and prospective) studies, it does not reflect real-world clinical environments, which are dynamically changing. Nevertheless, current standards require re-approval of AI software each time the model is adapted during deployment, thereby potentially allowing to adjust for such scenarios. While continual machine learning may be another promising solution to changes at unknown timepoints, it is currently unclear when and to which extent continual learning strategies will be implemented for applications in medicine [46]. Hence, to facilitate studies with large-scale and technical heterogenous cohorts, as well as to accelerate clinical translation, harmonization approaches will play an important role in the near future.

In summary, we present here a GAN-harmonization method that has the potential to improve the reproducibility and predictive performance of quantitative PET imaging. We demonstrated the ability of GAN-harmonization to enhance predictive performance by directly linking PET image harmonization to an improved clinical outcome prediction for HN cancer patients.

### Supplementary Information

Below is the link to the electronic supplementary material.

Supplementary file1 (PDF 829 KB)

## Data Availability

A whole-body FDG-PET/CT dataset with manually annotated tumor lesions (FDG-PET-CT-Lesions): 10.7937/gkr0-xv29 (TCIA) Head-Neck-PET-CT: 10.7937/K9/TCIA.2017.8oje5q00 (TCIA)

## Abbreviations

CycleGAN: Cycle-consistent generative adversarial network
LC: Lung cancer
HN: Head and neck
DM: Distant metastasis
First-order: First-order statistics
GLCM: Gray Level Cooccurrence Matrix
GLRLM: Gray Level Run Length Matrix
GLSZM: Gray Level Size Zone Matrix
GLDM: Gray Level Dependence Matrix
NGTDM: Neighboring Gray Tone Difference Matrix
COV: Coefficient of variation

## Code availability

The relevant code that supports the findings of this study is publicly available at GitHub: https://github.com/dhaberl/3DCycleGAN.
