# Supplementary material for: Multicenter PET image harmonization using generative adversarial networks
Source: Eur J Nucl Med Mol Imaging. 2024 May 2;51(9):2532–46. doi: 10.1007/s00259-024-06708-8 (PMC11224088; doi:10.1007/s00259-024-06708-8)
Supplement: Supplementary file 1 — Supplementary file1 (PDF 829 KB) [file 259_2024_6708_MOESM1_ESM.pdf]

Multicenter PET Image Harmonization using Generative Adversarial Networks

SUPPLEMENTARY MATERIAL

A. Model architecture, training, and inference details

Preprocessing

PET image intensities were converted to standardized uptake values (SUV) normalized to body weight. Based on previous experiments, we found that irregularities in the intensity distribution can potentially confound the learning task as the GAN may overweight or even overfit to these deviations. We therefore performed intensity normalization steps that helped to regularize the intensity distribution of each image resulting in a more stable training and higher quality results. We found that background noise in air (outside the body contour) was often leading to image artifacts as individual signal-containing voxels in air - that are mainly surrounded by signalless-voxels - would increase the complexity of the image generation learning task. We suppressed this background noise by applying a global threshold to the SUV images of 0.1 SUV units, effectively setting all intensities below this threshold to zero. Furthermore, we found that very high intensity peaks in individual voxels, typically observed at the injection site or in the bladder, can also cause artifacts in the generated output images. We treated those intensity peaks as outliers and bypassed them by clipping the image intensities to the 99.9-th percentile of all intensities found in the foreground voxels, which are defined as all voxels with an intensity greater than zero. The resulting image intensities were then rescaled from SUV units to be in a range of [-1, +1] using a linear transformation. All preprocessing steps were performed on a per image level.

Training details

We trained our network from scratch for 1000 epochs as a trade-off between runtime and reward based on previous experiments. Since the training is unsupervised, no direct stopping criterion was

used. We set the batch size to 1 in favor of increased patch sizes during training as we wanted the contextual information to be maximized. The network's weights were updated by stochastic gradient descent using the Adam optimization algorithm with exponential decay rates of $\beta_1 = 0.5$ and $\beta_2 = 0.999$. We started training with an initial learning rate of 0.0002 and linearly decayed it to zero after reaching 500 epochs. In contrast to Zhu et al., we did not initialize weights from a Gaussian distribution. The adversarial loss was defined as the mean squared error (Supplementary S2 Equation 3), while cycle-consistency and identity loss were ensured by the residual error between the input image y and the generated output image $\hat{y}$:

$$\text{Residual error} = \sum_{\mathrm{i}=1}^{\mathrm{N}} \mid y_i - \hat{y}_i \mid.$$

No data augmentation techniques were performed to avoid interfering effects on the site-specific intensity distributions.

Inference and post-processing

Images at test time were transformed from SUV units to a scale between [-1, +1], and predicted using a sliding window with the same size as the patch size used during training. After the prediction, images were transformed back to SUV units.

B. Image quality metrics

We calculated the following metrics between the input image y and the generated output image $\hat{y}$ to assess the image quality after harmonization:

Structural similarity index measure (SSIM)

The SSIM is a perceptual-based image quality metric that not only considers image degradation as a perceived change in structural information $s(y,\hat{y})$, but also as a change in luminance $l(y,\hat{y})$ and contrast $c(y,\hat{y})$:

$$\mathrm{SSIM}(y,\hat{y}) = \mathrm{l}(y,\hat{y})^{\alpha} \cdot c(y,\hat{y})^{\beta} \cdot s(y,\hat{y})^{\gamma},$$

where α, β and γ are factors to weight the importance of each component. We calculated the mean SSIM, defined as the average SSIM over multiple patches using a window size of 7 and weighting factors of $\alpha, \beta, \gamma = 1$. A comprehensive explanation on the calculation of each component of the SSIM can be found in [1].

Normalized root mean squared error (NRMSE)

$$\mathrm{MSE} = \frac{1}{N}\sum_{\mathrm{i}=1}^{\mathrm{N}}(y_i - \hat{y}_i)^2$$

$$\mathrm{NRMSE} = \frac{\sqrt{\mathrm{MSE}}}{\sqrt{\frac{1}{\mathrm{N}}\sum_{\mathrm{i}=1}^{\mathrm{N}} y_i^2}}$$

Peak signal to noise ratio (PSNR)

$$\mathrm{PSNR} = 10 \cdot \log_{10}\left(\frac{R^2}{MSE}\right)$$

All metrics were computed using scikit-image 0.19.3 [2].

Supplementary Fig. 1 Deep learning architecture. (a) Generator and (b) Discriminator

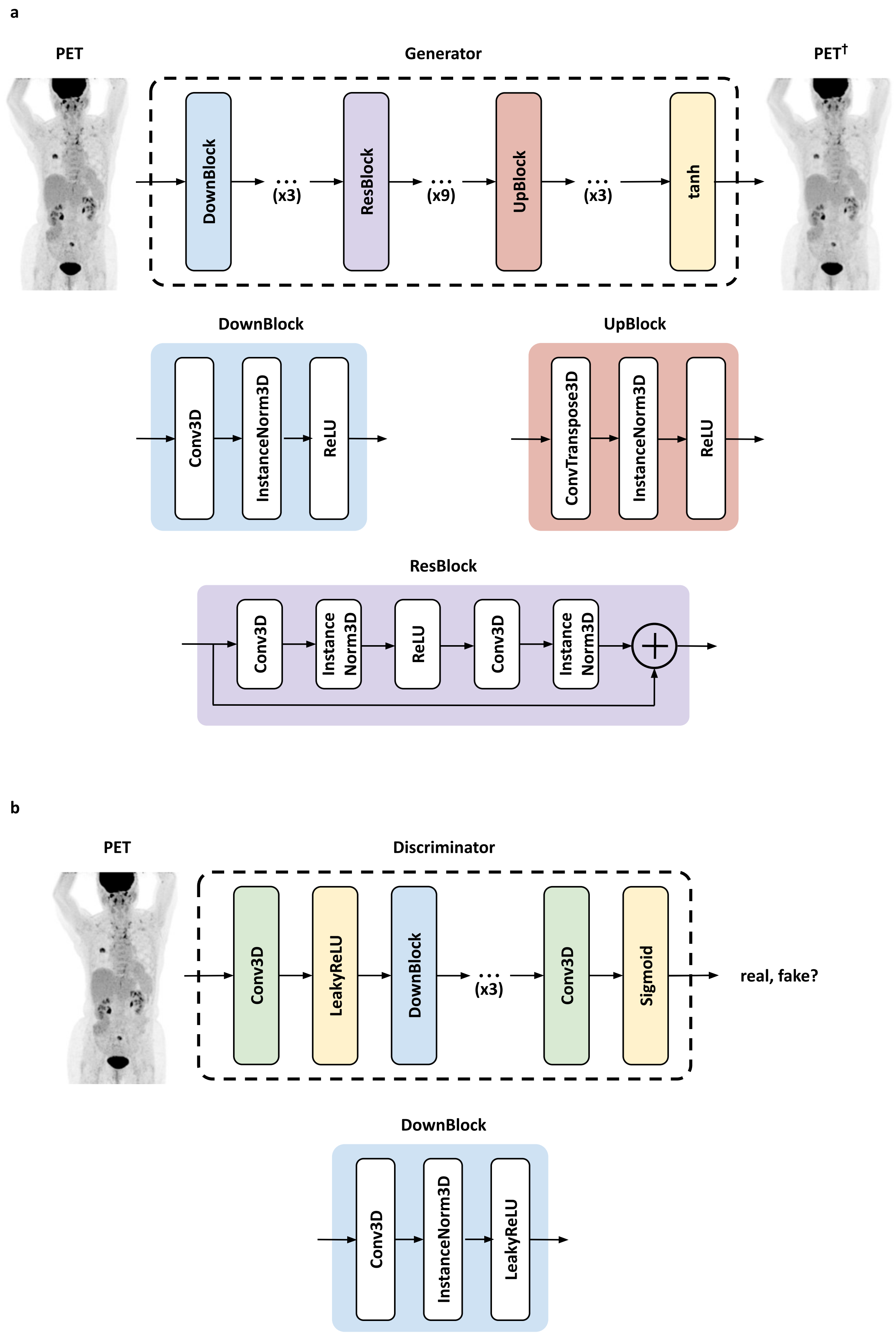

Supplementary Fig. 2 Feature distribution overlap. The overlap of two feature distributions (same feature but drawn from two different centers) is used as a measure of agreement. An overlap of 1 implies perfect agreement and thus high reproducibility, while an overlap of 0 corresponds to perfect disagreement and thus low reproducibility across the two centers

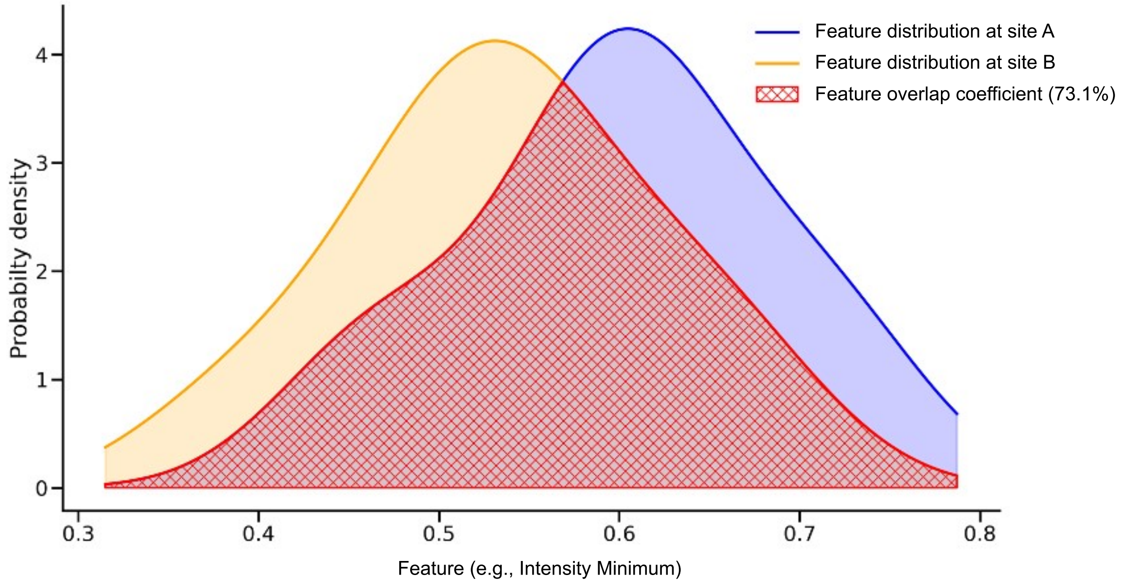

Supplementary Table 1 Radiomics reporting table

	Head and Neck dataset	Lung dataset
Region of interest	Largest lesion	Healthy liver tissue
Segmentation	see Vallières et al. (1)	Manually by a nuclear medicine physician using 3DSlicer software.
VOI definition	see Vallières et al. (1)	Spherical VOI with 3cm diameter placed in the upper right lobe of the healthy liver, refer to EANM procedure guideline version 2.0 (2)
Minimum VOI size	64 voxels as recommended in (3)	
Data type	Standardized uptake values normalized to body weight (SUV)	
Bin width	0.5	
Intensity discretization	Starting with zero (0 SUV)	
Interpolation method	B-Spline (PET image), Nearest-neighbor (Segmentation mask)	
Voxel size (mm^3)	1.5 × 1.5 × 1.5	3 × 3 × 3
Software package	PyRadiomics 3.0.1 (4)	
Features	First-order – First-order statistics (n=18)<br>GLCM – Gray Level Cooccurrence Matrix (n=24)<br>GLRLM – Gray Level Run Length Matrix (n=16)<br>GLSZM – Gray Level Size Zone Matrix (n=16)<br>GLDM – Gray Level Dependence Matrix (n=14)<br>NGTDM – Neighbouring Gray Tone Difference Matrix (n=5)	

Supplementary Table 2 Quantitative results for image similarity and image quality metrics between the original input images and their GAN-harmonized counterparts for the head and neck dataset. The reported metrics are computed globally from the entire body. Data are reported as mean values ± one standard deviation over all samples in a center. SSIM – Structural similarity, NRMSE – Normalized root mean squared error, PSNR – Peak signal to noise ratio

**Dataset**	**Harmonization direction**	**SSIM**	**NRMSE**	**PSNR**
Head and Neck	HGJ	0.969 ± 0.005	0.070 ± 0.026	35.894 ± 3.411
	CHUM-HMR	0.954 ± 0.033	0.085 ± 0.042	35.301 ± 3.709

Supplementary Table 3 Top 10 most contributing features as calculated from the mean feature importances over 100-folds. Results are shown for the harmonization from CHUM-HMR to HGJ (reference site: HGJ)

Harmonization method	Feature class	Feature name	Mean	SD
None	GLSZM	GrayLevelNonUniformity	6.96	0.68
	First-order statistics	Entropy	4.53	0.49
	GLDM	DependenceVariance	4.17	0.42
	First-order statistics	Skewness	3.78	0.42
	First-order statistics	Maximum	3.45	0.44
	NGTDM	Complexity	3.42	0.4
	GLSZM	GrayLevelVariance	3.17	0.39
	First-order statistics	Range	3.15	0.41
	GLDM	DependenceNonUniformity	2.87	0.39
	First-order statistics	Median	2.77	0.36
GAN	GLSZM	GrayLevelNonUniformity	7.78	0.7
	First-order statistics	Maximum	5.08	0.55
	First-order statistics	Range	4.92	0.53
	GLCM	InverseVariance	4.38	0.45
	GLDM	SmallDependenceHighGrayLevelEmphasis	4.16	0.45
	GLCM	MaximumProbability	3.43	0.37
	GLSZM	ZoneEntropy	3.14	0.37
	GLSZM	SmallAreaHighGrayLevelEmphasis	3.05	0.37
	GLSZM	SizeZoneNonUniformity	2.77	0.36
	GLDM	GrayLevelNonUniformity	2.56	0.37
ComBat	GLSZM	GrayLevelNonUniformity	6.34	0.73
	GLDM	DependenceVariance	4.17	0.47
	First-order statistics	Entropy	4.0	0.5
	NGTDM	Complexity	3.76	0.46
	GLCM	MaximumProbability	3.09	0.39
	GLDM	GrayLevelNonUniformity	3.07	0.45

	First-order statistics	Maximum	3.03	0.42
	First-order statistics	Range	3.0	0.42
	GLRLM	GrayLevelNonUniformity	2.81	0.49
	GLDM	DependenceNonUniformity	2.45	0.39
GAN and ComBat	First-order statistics	Maximum	5.33	0.63
	First-order statistics	Range	4.6	0.58
	NGTDM	Complexity	3.97	0.52
	First-order statistics	RobustMeanAbsoluteDeviation	3.84	0.49
	GLDM	GrayLevelNonUniformity	3.52	0.53
	GLCM	DifferenceVariance	3.26	0.45
	GLDM	LargeDependenceLowGrayLevelEmphasis	3.06	0.53
	GLSZM	GrayLevelNonUniformity	3.0	0.49
	First-order statistics	Entropy	2.95	0.45
	GLSZM	GrayLevelNonUniformityNormalized	2.83	0.41

Supplementary Table 4 Top 10 most contributing features as calculated from the mean feature importances over 100-folds. Results are shown for the harmonization from HGJ to CHUM-HMR (reference site: CHUM-HMR)

Harmonization method	Feature class	Feature name	Mean	SD
None	GLSZM	GrayLevelNonUniformity	6.96	0.68
	First-order statistics	Entropy	4.53	0.49
	GLDM	DependenceVariance	4.17	0.42
	First-order statistics	Skewness	3.78	0.42
	First-order statistics	Maximum	3.45	0.44
	NGTDM	Complexity	3.42	0.4
	GLSZM	GrayLevelVariance	3.17	0.39
	First-order statistics	Range	3.15	0.41
	GLDM	DependenceNonUniformity	2.87	0.39
	First-order statistics	Median	2.77	0.36
GAN	GLSZM	GrayLevelNonUniformity	5.17	0.62
	GLDM	DependenceVariance	4.44	0.47
	First-order statistics	Entropy	3.94	0.49
	First-order statistics	Maximum	3.51	0.46
	NGTDM	Complexity	3.4	0.43
	First-order statistics	Skewness	3.33	0.41
	First-order statistics	Range	3.13	0.42
	GLSZM	GrayLevelNonUniformityNormalized	2.76	0.37
	GLDM	LargeDependenceLowGrayLevelEmphasis	2.51	0.47
	GLRLM	ShortRunLowGrayLevelEmphasis	2.39	0.37
ComBat	GLSZM	GrayLevelNonUniformity	6.32	0.73
	GLDM	DependenceVariance	4.19	0.47
	First-order statistics	Entropy	3.99	0.5
	NGTDM	Complexity	3.76	0.46
	GLCM	MaximumProbability	3.06	0.39
	GLDM	GrayLevelNonUniformity	3.02	0.45

	First-order statistics	Maximum	3.0	0.42
	First-order statistics	Range	2.96	0.41
	GLRLM	GrayLevelNonUniformity	2.8	0.5
	GLDM	DependenceNonUniformity	2.47	0.39
GAN and ComBat	GLSZM	GrayLevelNonUniformity	8.23	0.88
	GLDM	SmallDependenceHighGrayLevelEmphasis	4.1	0.53
	GLCM	MaximumProbability	4.01	0.49
	First-order statistics	Range	3.56	0.49
	First-order statistics	Maximum	3.26	0.47
	GLSZM	SizeZoneNonUniformity	3.2	0.47
	GLDM	GrayLevelNonUniformity	2.7	0.47
	GLDM	DependenceNonUniformity	2.38	0.38
	First-order statistics	RobustMeanAbsoluteDeviation	2.32	0.35
	NGTDM	Complexity	2.16	0.34

REFERENCES

1. Vallières M, Kay-Rivest E, Perrin LJ, Liem X, Furstoss C, Aerts HJWL, et al. Radiomics strategies for risk assessment of tumour failure in head-and-neck cancer. Sci Rep [Internet]. 2017 Aug 31;7(1):10117. Available from: https://www.nature.com/articles/s41598-017-10371-5
2. Boellaard R, Delgado-Bolton R, Oyen WJG, Giammarile F, Tatsch K, Eschner W, et al. FDG PET/CT: EANM procedure guidelines for tumour imaging: version 2.0. Eur J Nucl Med Mol Imaging [Internet]. 2015 Feb 2;42(2):328–54. Available from: http://link.springer.com/10.1007/s00259-014-2961-x
3. Orlhac F, Nioche C, Klyuzhin I, Rahmim A, Buvat I. Radiomics in PET Imaging. PET Clin [Internet]. 2021 Oct;16(4):597–612. Available from: https://linkinghub.elsevier.com/retrieve/pii/S1556859821000468
4. van Griethuysen JJM, Fedorov A, Parmar C, Hosny A, Aucoin N, Narayan V, et al. Computational Radiomics System to Decode the Radiographic Phenotype. Cancer Res [Internet]. 2017 Nov 1;77(21):e104–7. Available from: https://aacrjournals.org/cancerres/article/77/21/e104/662617/Computational-Radiomics-System-to-Decode-the
